# Recovery, Isolation, and Characterization of Food Proteins

**DOI:** 10.3390/foods11010070

**Published:** 2021-12-29

**Authors:** Ute Schweiggert-Weisz, Emanuele Zannini

**Affiliations:** 1 Institute for Nutritional and Food Sciences, University of Bonn, 53113 Bonn, Germany; 2 Department Food Process Development, Fraunhofer Institute for Process Engineering and Packaging (IVV), 85354 Freising, Germany; 3 Cereal and Beverage Science Research Group, School of Food and Nutritional Sciences, University College Cork, College Road, T12K8AF Cork, Ireland; e.zannini@ucc.ie

One of the greatest challenges currently facing our society is combating climate change. The agricultural and food sectors are critical, as they are responsible for a considerable share of greenhouse gas emissions. Increasing competition for available resources emphasises the need to change consumption and food production styles. This is the only way to continue to have enough raw materials to produce food for a growing world population.

Reducing the production of animal proteins and replacing them with plant proteins could make an essential contribution to a more sustainable diet. For this reason, the recovery of functional proteins from plant raw materials or by-products of food production is currently an essential and strategic research topic in both food science and the food industry. Protein production is important, but so is the characterisation of the products’ properties, such as their sensory or physicochemical behaviours. Only those protein products with good taste and high functionality will have long-term success in the market. 

This Special Issue belongs to the section “Food Physics and (Bio)Chemistry”, and is focused on the utilisation of protein-rich raw materials and by-products from the food industry to produce food ingredients and food products. It also shows how the functional properties of proteins can be improved. This Special Issue includes seven manuscripts, six being research papers and one a review paper, all of which are important contributions to this topic made by distinguished experts in this area.

Manuscript 1 is titled “Rejuvenated Brewer’s Spent Grain: EverVita Ingredients as Game-Changers in Fibre-Enriched Bread” [1]. It describes the use of brewer’s spent grain —the main side-stream of brewing—as a raw material for producing functional protein and dietary fibre-rich ingredients. The study investigated the impact of two Brewer’s spent grain ingredients on dough and bread quality, and the nutritional value of the bread. The dietary fibre-rich ingredient had almost no influence on dough and bread quality. However, the protein-rich ingredient significantly improved the nutritional value of the bread by increasing the protein content by 36%. Thus, both ingredients could be game changers in developing bread fortified with dietary fibres and proteins.

Manuscript 2 (“Extrusion Processing of Rapeseed Press Cake-Starch Blends: Effect of Starch Type and Treatment Temperature on Protein, Fiber and Starch Solubility”) [2] is a fascinating study on the behaviour of a protein-rich rapeseed press cake in a starch matrix during extrusion. In the extruded blends, the starch type significantly impacted protein solubility, which decreased with increasing barrel temperature. These effects could be attributed to the inconsistent process conditions, due to the different rheological properties of the starches, rather than to molecular interactions of the starches with the rapeseed proteins. Since texturates play an important role in the production of meat substitutes, these results can contribute important insights into the behaviour of the proteins in the end product, and possibly also to their nutritional evaluation.

Manuscript 3 “Screening of Twelve Pea (*Pisum sativum* L.) Cultivars and their Isolates Focusing on the Protein Characterization, Functionality, and Sensory Profiles” is dedicated to a raw material currently in great demand for the production of protein isolates and concentrates—peas (*Pisum sativum* L.) [3]. The authors investigated the suitability of twelve pea cultivars for the production of protein ingredients. While the protein yield differed between the cultivars, their proximate composition and physicochemical properties were quite similar. However, there were some differences in sensory properties in particular in the pea-like aroma attribute and the bitter taste.

Manuscript 4 entitled “Assessment of the Physicochemical and Conformational Changes of Ultrasound-driven Proteins Extracted from Soybean Okara Byproduct” is a valuable work about the utilization of ultrasound to extract proteins from okara—the by-product of soy food manufacturing [4]. Besides the protein yields and protein solubility, changes in the protein structure and an enhanced peptide generation were observed. Therefore, the okara protein extracted through the ultrasound technique is a valuable raw material for new applications. 

Manuscript 5 (“Functional Properties of Rye Prolamin (Secalin) and their Improvement by Protein Lipophilization through Capric Acid Covalent Binding”) deals with the modification of the prolamin fraction of rye protein to improve its functional properties [5]. Using lyophilization, the protein solubility and water absorption capacity decreased, but an increase in oil absorption, emulsifying/foaming capacity and stability was observed.

An improvement of the functional properties of lupin protein isolates using enzymatic hydrolysis followed by lactic fermentation is shown in Chapter 6, entitled “Fermentation of Lupin Protein Hydrolysates—Effects on their Functional Properties, Sensory Profile and the Allergenic Potential of the Major Lupin Allergen Lup an 1” [6]. In particular, the protein solubility and foaming activity at pH 4.0 increased significantly. In addition, Bead-Assay showed a successful reduction of the allergen fraction Lup an 1.

Manuscript 7 contains a review entitled “Barley Protein Properties, Extraction and Applications, with a Focus on Brewers Spent Grain Protein” [7]. This manuscript is the only review in this Special Issue/book, and excellently completes this book by giving a comprehensive overview of barley and brewers’ spent grain proteins. The review explores the extraction and application of proteins from these raw materials, and offers insights into the protein composition. Brewers spent grains are a by-product of the brewery industry that is produced in large quantities. The utilisation of brewers’ spent grains is thus an excellent example of the complete utilization of agricultural raw materials for sustainable nutrition.

The manuscripts in this book deal with the undeniable importance of protein ingredients for the food industry. At the same time, they show the diverse research opportunities that the recovery, isolation, and characterisation of food protein entail. Proteins can be extracted from plant materials with tailor-made functional properties. In addition, the utilisation of by-products of food production in food production offers the possibility for the holistic use of our agricultural products, with maximum exploitation of our rural resources. Both strategies—the increased cultivation and processing of protein-rich raw materials and the increased utilisation of agricultural products—offer promising ways to make our consumption and production styles more sustainable in the future.
